# Blimp-1 orchestrates macrophage polarization and metabolic homeostasis via purine biosynthesis in sepsis

**DOI:** 10.1038/s41419-025-07405-6

**Published:** 2025-02-06

**Authors:** Wenjuan Peng, Qiushi Qin, Rui Li, Yujia Liu, Lan Li, Yue Zhang, Liuluan Zhu

**Affiliations:** 1 Beijing Key Laboratory of Emerging Infectious Diseases, Institute of Infectious Diseases, Beijing Ditan Hospital, Capital Medical University, Beijing, 100015 China; 2 Beijing Institute of Infectious Diseases, Beijing, 100015 China; 3 National Center for Infectious Diseases, Beijing Ditan Hospital, Capital Medical University, Beijing, 100015 China; 4 Institute of Infectious Diseases, Peking University Ditan Teaching Hospital, Beijing, 100015 China

**Keywords:** Monocytes and macrophages, Infectious diseases

## Abstract

Sepsis is a life-threatening condition characterized by a dysregulated immune response to infection, leading to systemic inflammation and organ dysfunction. Macrophage polarization plays a critical role in pathogenesis of sepsis, and the influence of B lymphocyte-induced maturation protein-1 (Blimp-1) on this polarization is an underexplored yet pivotal aspect. This study aimed to elucidate the role of Blimp-1 in macrophage polarization and metabolism during sepsis. Using a murine cecal ligation and puncture model, we observed elevated Blimp-1 expression in M2 macrophages. Knockdown of Blimp-1 by macrophage-targeted adeno-associated virus in this model resulted in decreased survival rates, exacerbated tissue damage, and impaired M2 polarization, underscoring its protective role in sepsis. In vitro studies with bone marrow-derived macrophage (BMDM), RAW264.7, and THP-1 cells further demonstrated Blimp-1 promotes M2 polarization and modulates key metabolic pathways. Metabolomics and dual-luciferase assays revealed Blimp-1 significantly influences purine biosynthesis and the downstream Ornithine cycle, which are essential for M2 macrophage polarization. In vitro studies with BMDM further suggested that the purine biosynthesis and Ornithine cycle metabolic regulation is involved in Blimp-1’s effects on M2 macrophage polarization, and mediates Blimp-1’s impact on septic mice. Our findings unveil a novel mechanism by which Blimp-1 modulates macrophage polarization through metabolic regulation, presenting potential therapeutic targets for sepsis. This study highlights the significance of Blimp-1 in orchestrating macrophage responses and metabolic adaptations in sepsis, offering valuable insights into its role as a critical regulator of immune and metabolic homeostasis.

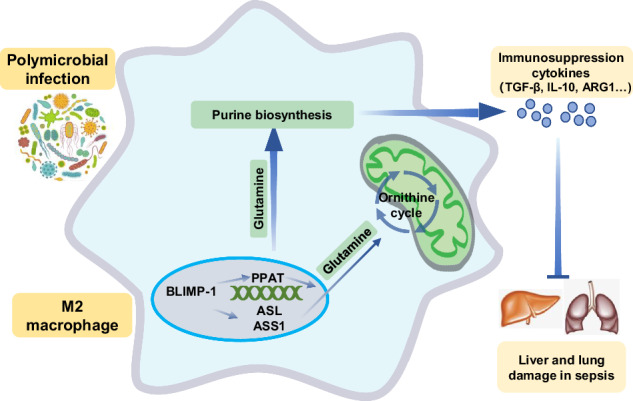

## Introduction

Sepsis, a life-threatening condition characterized by organ dysfunction resulting from a dysregulated host response to infection, affects approximately 48.9 million individuals globally each year [1]. In 2017, it was responsible for 11 million deaths, accounting for 19.7% of all global mortality [2]. The pathogenesis of sepsis is multifaceted, involving intricate interactions between infectious microorganisms and the host, alongside various pathways including infection, inflammation, hypoxia, and metabolic reprogramming [3]. A key mechanism underpinning sepsis is the imbalance of inflammatory response. During the early stages of sepsis, the host’s immune response is highly inflammatory, with numerous inflammatory mediators present that damage both tissues and immune cells. This is followed by an advanced stage of sepsis, characterized by host immunosuppression [4]. Consequently, the primary therapeutic goal for sepsis patients is the regulation of staging immunophenotypes and personalized immune therapies, which may effectively reduce sepsis-related mortality.

In sepsis-induced organ dysfunction, monocytes and macrophages are the first line of host defense. Recent study indicates that circulation-derived Ly6C^hi^ inflammatory monocytes are recruited to the inflamed livers where they differentiated into macrophages. These monocyte-derived macrophages can even replace tissue macrophages, directly inducing or exacerbating tissue injury [5]. Macrophages play a crucial role in mediating inflammatory responses and primarily contain two functional subsets: pro-inflammatory (M1) and anti-inflammatory (M2) macrophages. In the early stage of sepsis, IFN-γ and LPS induce M1 macrophage polarization [6, 7]. The increasing number of M1 macrophages continue to release large quantities of inflammatory factors, such as IL-1β, TNF-α, and IL-6 [6, 7], leading to severe inflammatory responses and subsequent organ damage. Conversely, during late-stage sepsis, M2 macrophages can be activated by IL-4 and IL-13 [8]. An excessive increase in M2 macrophages results in the secretion of large quantities of IL-10, TGF-β, and other anti-inflammatory cytokines, inducing an immunosuppressive state in the host [6, 7, 9]. This leads to immune paralysis and the recurrence of severe infection. Given the critical role of the M1/M2 polarization in sepsis [10], it is essential to understand the regulatory mechanisms governing macrophage polarization. This is crucial for elucidating sepsis pathology and exploring new immunotherapy modalities.

B lymphocyte-induced maturation protein-1 (Blimp-1) is a transcription suppressor of IFN-β encoded by the *Prdm1* gene [11, 12]. Overexpression of Blimp-1 in pro-monocytic cells induces partial macrophage differentiation, characterized by the expression of surface markers such as CD11b and CD11c [13]. Blimp-1 was identified as a marker for a specific macrophage population located near the microbe-exposed surface in the colon [14]. Recent studies have shown CCL8 is a direct target of Blimp-1 in mononuclear macrophages, with elevated CCL8 levels observed in *Prdm1*^fl^/^fl^ mice [15]. Our previous study claimed Blimp-1 represses the production of several inflammatory cytokines, including IL-1β, IL-6, and IL-18, by directly binding to the genomic region and restricting the nuclear translocation and transcriptional activity of NF-κB [16]. However, the potential regulatory role of Blimp-1 in macrophage polarization and its significance in sepsis represent a critical gap in current understanding.

Emerging evidence indicates energy metabolism plays a crucial role in regulating macrophage polarization and function [17]. Generally, pro-inflammatory M1 macrophages primarily rely on glycolysis to generate energy for innate immune responses, while M2 macrophages depend more on mitochondrial oxidative phosphorylation and the tricarboxylic acid cycle to satisfy their energy requirements [18]. During M2 polarization, transcription factors such as PPARγ and PGC-1β are critical for mitochondrial respiration and fatty acid oxidative metabolism [19, 20]. Additionally, transketolase (TKT) is important for glycometabolism [21], while glutamate-pyruvate transaminase (GPT) and glycine amidinotransferase (GATM) are involved in amino acid metabolism [22]. Notably, Blimp-1 may participate in cellular metabolism, thereby modulating inflammatory marker expression in quiescent macrophages [23]. For instance, Blimp-1 regulates IL-10 expression in group 2 innate lymphoid cells (ILC2s), which exhibit a metabolic dependence on glycolysis for IL-10 production [24]. These findings suggest that Blimp-1 may regulate macrophage polarization through cellular metabolic pathways, thus influencing inflammatory responses in sepsis.

In this study, we aim to elucidate the effects of Blimp-1 on macrophage polarization in sepsis from the perspective of cellular metabolism. We inhibited Blimp-1 expression and measured its effects on macrophage polarization phenotypes and functions both in vitro and in vivo. Subsequently, we utilized the metabolomics technology to explore the metabolic mechanisms by which Blimp-1 regulates macrophage polarization. We conducted dual-luciferase reporter assay to validate the metabolic pathways regulated by Blimp-1 in macrophages. In summary, our findings clarify a previously unknown mechanism by which Blimp-1 plays a key role in orchestrating macrophage polarization during sepsis.

## Results

### Blimp-1 expression is elevated in sepsis-associated macrophages

**Fig. 1:**
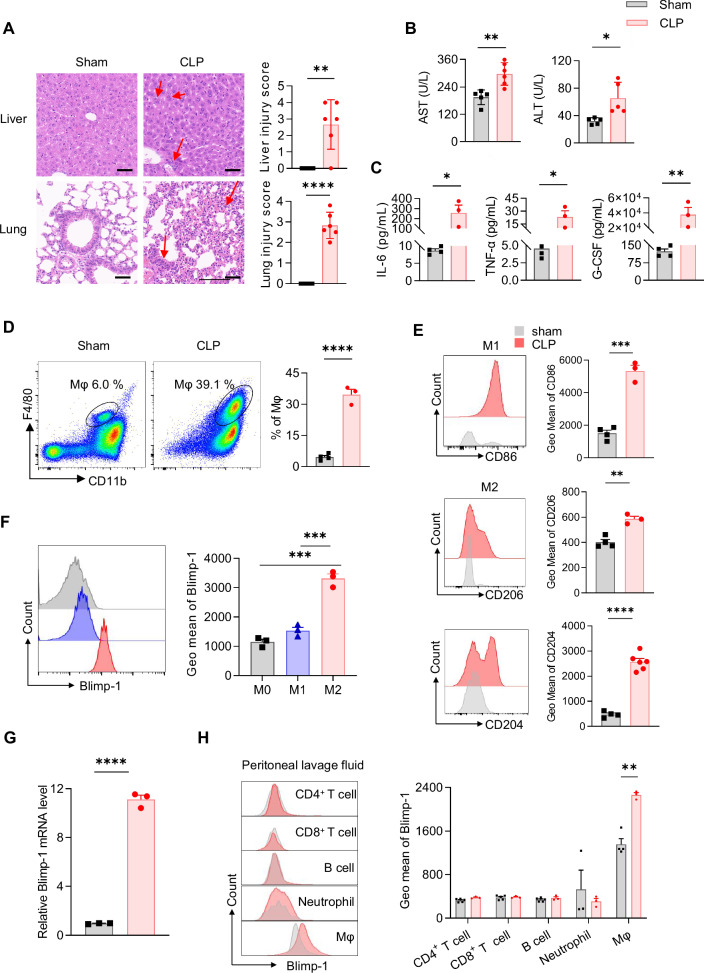
Blimp-1 expression was significantly increased in macrophages in CLP mice. **A** H&E staining of liver and lungs in CLP and sham mice. The liver tissue displayed a disordered lobular structure, swollen and deformed hepatocytes, and dispersed binuclear hepatocytes (red arrow). The alveolar space was widened with inflammatory cells infiltration and substantial leakage of red blood cells (red arrow). Scale bar = 50 μm. The degree of liver injury was evaluated according to Ishak score. The degree of lung injury was evaluated based on pulmonary hemorrhage, pulmonary edema and widening of pulmonary septum. **B** Serum levels of ALT and AST. **C** Serum levels of inflammatory cytokines. **D** Percentage of macrophages in peritoneal lavage fluid (PLF), assessed by flow cytometry. **E** Geometric mean of M1 (CD86^+^) and M2 (CD206^+^ or CD204^+^) macrophages in PLF, assessed by flow cytometry. **F** Geometric mean of Blimp-1 in macrophages with different polarization phenotypes in PLF, analyzed by flow cytometry. **G** The mRNA levels of *Blimp-1* in PLF cells, measured by real-time PCR. **H** Geometric mean of Blimp-1 in CD4^+^ T cells, CD8^+^ T cells, B cells, neutrophils and macrophages in the PLF of mice. Data are presented as the mean ± standard error of independent experiments. *P < 0.05, **P < 0.01, ***P < 0.001, ****P < 0.0001.

We had successfully established a cecal ligation and puncture (CLP) sepsis model in C57BL/6 J mice. Compared to sham-operated controls, CLP mice exhibited significant histopathological lesions in liver and lungs, along with increased serum levels of hepatic injury biomarkers and inflammatory cytokines (Fig. 1A–C). Importantly, the peritoneal lavage fluid (PLF) of CLP mice contained a higher percentage of macrophages, with Blimp-1 expression specifically elevated in the M2 subset (Fig. 1D–F). The mRNA level of *Blimp-1* was also significantly increased in these cells (Fig. 1G). This selective upregulation of Blimp-1 was observed in NK cells, γδ T cells and M2 macrophages, but not in dendritic cells, CD4^+^ T cells, CD8^+^ T cells, B cells or neutrophils (Fig. 1H, Fig. S1A-1C). Given that dendritic cells, NK cells, and γδT cells constitute a minor proportion of immune cells in the mouse peritoneal cavity, it suggests that Blimp-1 is predominantly expressed in M2 macrophages within PLF and plays a vital role in sepsis (Fig. S1D).

### Blimp-1 knockdown exacerbates sepsis by impairing M2 polarization

**Fig. 2:**
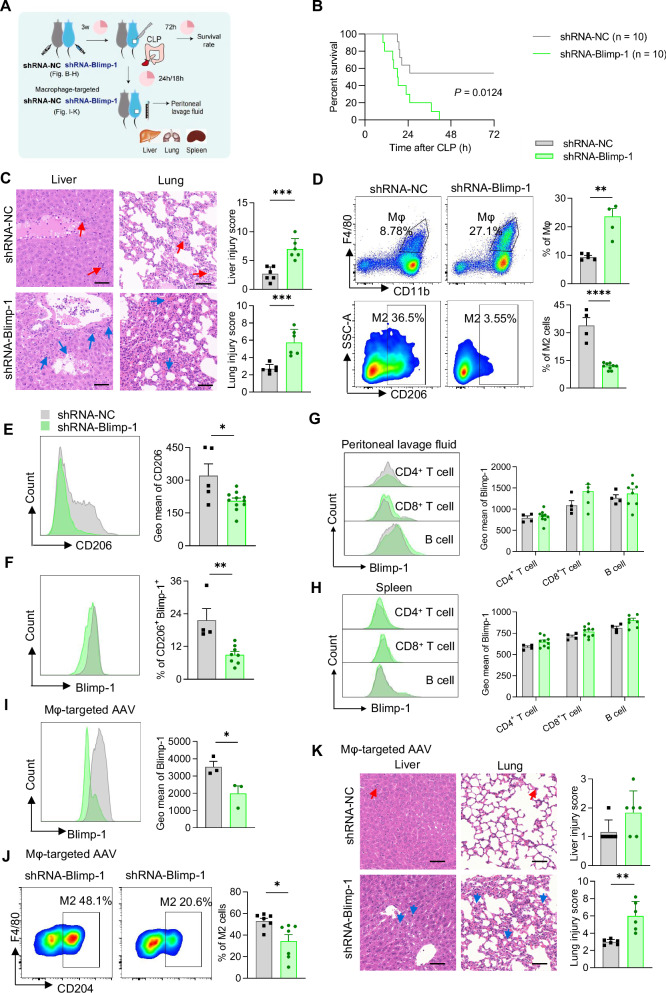
ShRNA-Blimp-1 aggravated multiple organ damages in CLP mice. **A** Diagram of CLP mouse model construction and experiment timeline. **B** The survival rates of CLP mice intraperitoneally injected with adeno-associated virus (AAV) carrying shRNA-NC or shRNA-Blimp-1, respectively (*n* = 10 per group). **C** H&E staining of liver and lungs in mice. Scale bar = 50 μm. Red arrows indicate hepatic lobular structure damage, inflammatory cell infiltration and aggregation, and hepatocyte swelling and deformation in the liver. Blue arrows indicate destroyed alveolar cavity with inflammatory cell infiltration and congestion in the lungs. The degree of liver injury was evaluated according to the Ishak score. The degree of lung injury was evaluated based on pulmonary hemorrhage, pulmonary edema and widening of pulmonary septum. **D** Percentage of total macrophages and M2 macrophages in PLF. **E** Geometric mean of CD206 in macrophages from PLF. **F** Percent of CD206^+^Blimp-1^+^ macrophages in PLF. **G** Geometric mean of Blimp-1 in CD4^+^ T cells, CD8^+^ T cells, and B cells from PLF. **H** Geometric mean of Blimp-1 in CD4^+^ T cells, CD8^+^ T cells, and B cells in the spleen. **I** Geometric mean of Blimp-1 in macrophage from PLF. **J** Percentage of M2 macrophages in PLF. **K** H&E staining of liver and lungs in mice. The alveolar space was widened with inflammatory cells infiltration and substantial leakage of red blood cells (red arrow). Scale bar = 50 μm. The degree of lung injury was evaluated based on pulmonary hemorrhage, pulmonary edema and widening of pulmonary septum. Data are presented as the mean ± standard error of independent experiments. *P < 0.05, **P < 0.01, ***P < 0.001, ****P < 0.0001. Mice are intraperitoneally injected with AAV particles carrying shRNA-Blimp-1 or shRNA-NC (**B**–**H**) and macrophage-targeted shRNA-Blimp-1 or shRNA-NC (**I**–**K**).

To elucidate the functional significance of Blimp-1 upregulation, we knocked down its expression using shRNA-Blimp-1 and assessed the impact on CLP mice (as depicted in Fig. 2A). Adeno-associated virus (AAV) effectively reduced Blimp-1 in mouse PLF cells (Fig. S2A). We also found shRNA released into the circulation (Fig. S2B). Three weeks post-injection, the survival rate of CLP mice was significantly lower in the Blimp-1 knockdown group (Fig. 2B). Histopathological analysis revealed exacerbated liver and lung tissue damage (Fig. 2C). These findings underscore the protective role of Blimp-1 in modulating the inflammatory response and tissue repair during sepsis. Following shRNA-Blimp-1 intervention, the PLF of CLP mice exhibited an elevated total macrophage count, yet a diminished proportion of M2 macrophages, as evidenced by reduced presence of CD206^+^ and CD206^+^Blimp-1^+^ cells (Fig. 2D–F). In contrast, Blimp-1 expression in other immune cells, such as CD4^+^ T cells, CD8^+^ T cells, and B cells, remained unaffected in both PLF (Fig. 2G) and spleen grinding fluid (Fig. 2H). These observations suggest that Blimp-1’s protective effect in sepsis may depend on its regulatory role in M2 macrophage polarization.

To establish that the observed effects of Blimp-1 in septic mice are specifically mediated by macrophages, we utilized a macrophage-targeted AAV to knock down its expression, effectively silencing Blimp-1 in macrophages (Fig. 2I). Mice injected with AAV exhibited expression of GFP in the liver and lung (Fig. S2C). Subsequent to this intervention with shRNA-Blimp-1 targeted at macrophages, the PLF from CLP mice exhibited a reduced proportion of M2 macrophages (Fig. 2J). We opted to conduct our time point analysis at an early stage, specifically at 18 hours, prior to any recorded animal fatalities. This early assessment might explain why histopathological analyses did not reveal liver tissue damage; however, an increase in lung tissue damage was distinctly evident (Fig. 2K).

### Blimp-1 promotes monocyte-macrophage differentiation and M2 polarization

**Fig. 3:**
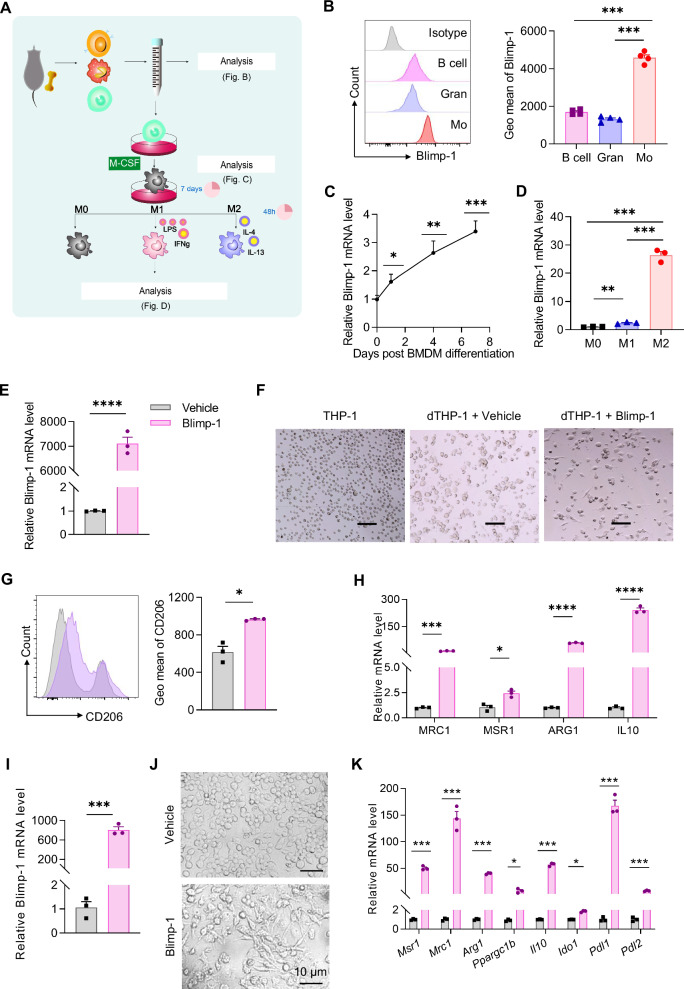
Overexpression of Blimp-1 promoted macrophage differentiation and M2 polarization. **A** Diagram of experimental process of bone marrow-derived macrophage (BMDM) differentiation and polarization. **B** Geometric mean of Blimp-1 in B cells, granulocytes, and monocytes from bone marrow of naïve mice. **C** The mRNA levels of *Blimp-1* during BMDM differentiation. **D** The mRNA levels of *Blimp-1* in different polarization types of BMDMs. **E** Morphology of THP-1 cells, differentiated THP-1 cells (dTHP-1), and differentiated THP-1 cells with Blimp-1 overexpression (dTHP-1 + bLIMP-1). Scale bar = 20 μm. **F** The mRNA levels of *Blimp-1* in dTHP-1 cells transfected with *Blimp-1* plasmid or vehicle. **G** Geometric mean of CD206 in dTHP-1 cells with Blimp-1 overexpression. **H** The mRNA levels of *MRC1, MSR1, ARG1* and *IL10* in dTHP-1 cells with Blimp-1 overexpression. **I** The mRNA levels of *Blimp-1* in RAW264.7 cells transfected with *Blimp-1* plasmid or vehicle. **J** Morphology of RAW264.7 cells. Scale bar = 10 μm. **K** The mRNA levels of M2 marker genes in RAW264.7 cells. Data are presented as the mean ± standard error of independent experiments. *P < 0.05, **P < 0.01, ***P < 0.001, ****P < 0.0001.

We next assessed the temporal expression of Blimp-1 during macrophage differentiation and polarization from bone marrow cells of naïve mice (as depicted in Fig. 3A). Blimp-1 expression was significantly higher in monocytes compared to B cells and granulocytes (Fig. 3B), and its mRNA levels progressively increased alongside bone marrow-derived macrophage (BMDM) differentiation and maturation (Fig. 3C). Upon polarization, M1 macrophages exhibited high secretion of IL-1β, IL-6, TNF-α, and IL-12 (Fig. S3A); whereas M2 macrophages secreted high levels of TGF-β, CCL17, and CCL22 (Fig. S3B). Notably, *Blimp-1* mRNA levels were specifically elevated in M2 macrophages compared to M0 and M1 counterparts (Fig. 3D), indicating a potential role for Blimp-1 in the differentiation and M2 polarization of macrophages.

To further dissect Blimp-1’s influence on macrophage polarization, THP-1 monocytic cells were induced to differentiate into macrophages and subsequently transfected with a *Blimp-1* expressing plasmid. The expression level of Blimp-1 was identified by real-time fluorescent quantitative PCR (Fig. 3E). Overexpression of Blimp-1 causes morphological changes consistent with M2 macrophages, including the appearance of extended pseudopods (Fig. 3F), and a significant increase in CD206 expression, as well as increased mRNA levels of M2 markers such as *MRC1, MSR1, ARG1* and *IL10* (Fig. 3G, H). Similar results were obtained with RAW264.7 macrophages overexpressing Blimp-1, where an increase in mRNA levels of *Msr1, Mrc1, Arg1, Ppargc1b, Il10, Ido1, Pdl1*, and *Pdl2* was observed (Fig. 3I–K). These findings collectively highlight the critical role of Blimp-1 in driving macrophage differentiation and the acquisition of the M2 phenotype.

### Blimp-1 is essential for energy metabolism in M2 macrophages

**Fig. 4:**
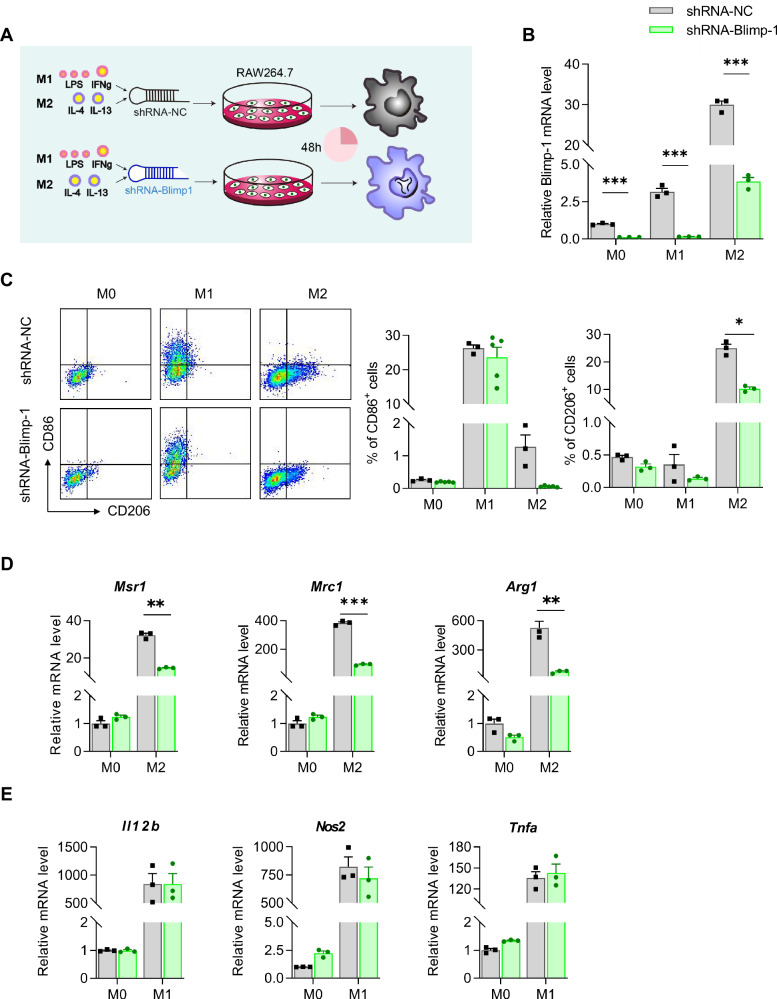
ShRNA-Blimp-1 attenuated M2 macrophage polarization. **A** Diagram of inducing polarization of RAW264.7 cells. **B** The mRNA levels of *Blimp-1* in macrophages infected with lentivirus carrying shRNA-NC or shRNA-Blimp-1. **C** Percentages of M1 and M2 macrophages. **D** The mRNA levels of *Mrc1, Msr1* and *Arg1* in M0 and M2 macrophages. **E** The mRNA levels of *Il12b, Nos2* and *Tnfa* in M0 and M1 macrophages. Data are presented as the mean ± standard error of independent experiments. *P < 0.05, **P < 0.01, ***P < 0.001, ****P < 0.0001.

Further investigation of Blimp-1’s regulatory role in macrophage polarization was conducted by knocking down its expression in RAW264.7 cell (Fig. 4A). Compared to scrambled non-specific control (shRNA-NC), shRNA-Blimp-1 effectively reduced Blimp-1 expression, particularly in M2 macrophages (Fig. 4B). This knockdown resulted in a significant decrease in the proportion of M2 (CD206^+^) macrophages and a dramatic reduction in the mRNA levels of M2 markers *Mrc1, Msr1*, and *Arg1* (Fig. 4C, D). In contrast, the proportion of M1 (CD86^+^) macrophages and the mRNA levels of M1 markers *Il12b, Nos2*, and *Tnfa* remained unchanged (Fig. 4C–E), underscoring the specific role of Blimp-1 in M2 macrophage polarization and function.

**Fig. 5:**
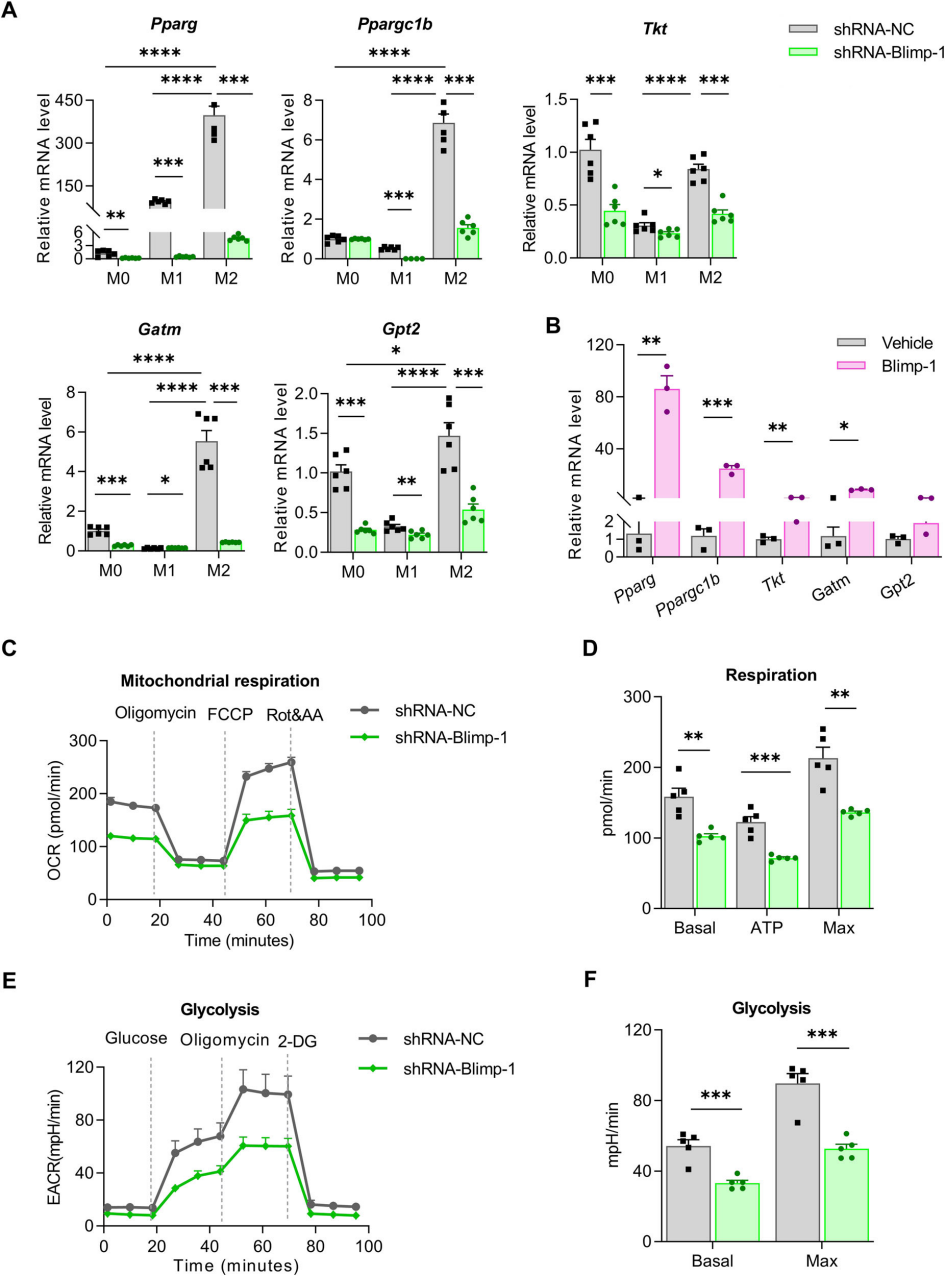
Blimp-1 regulated energy metabolism and metabolic marker expressions in M2 macrophages. **A** The mRNA levels of *Pparg, Ppargc1b, Tkt, Gatm* and *Gpt2* in M2 macrophages infected with lentivirus carrying shRNA-NC or shRNA-Blimp-1. **B** The mRNA levels of *Pparg, Ppargc1b, Tkt, Gatm* and *Gpt2* in RAW264.7 macrophages transfected with or without *Blimp-1* expressing plasmid. **C** Oxygen consumption rate (OCR) curve of the Seahorse Mito Stress Test in M2 macrophages infected with lentivirus carrying shRNA-NC or shRNA-Blimp-1. **D** Quantification of OCR parameters in M2 macrophages. **E** Extracellular acidification rate (ECAR) curve of the Seahorse Glycolytic Test in M2 macrophages infected with lentivirus carrying shRNA-NC or shRNA-Blimp-1. **F** Quantification of ECAR parameters in M2 macrophages. FCCP: Trifluoromethoxy carbonyl cyanide phenylhydrazone; Rot: rotenone; AA: antimycin A; 2-DG: 2-Deoxy-d-Glucose. Data are presented as the mean ± standard error of independent experiments. *P < 0.05, **P < 0.01, ***P < 0.001, ****P < 0.0001.

We also examined the expression of genes pivotal to mitochondrial respiration, fatty acid oxidative metabolism, and glycometabolism. The robust expression levels of *Pparg, Ppargc1b, Tkt, Gatm*, and *Gpt2* in M2 macrophages, which were notably diminished following Blimp-1 knockdown (Fig. 5A). Conversely, overexpression of Blimp-1 in RAW264.7 macrophages resulted in elevated mRNA levels for these genes (Fig. 5B). Employing the Seahorse XF analyzer, we conducted metabolic assays substantiated the impact of Blimp-1 on mitochondrial function. The knockdown of Blimp-1 corresponded with a reduction in mitochondrial oxygen consumption rates (OCRs) in M2 macrophages (Fig. 5C). This reduction is indicative of a decrease in the maximal respiration, mitochondrial ATP production, and spare respiratory capacity (Fig. 5D). Furthermore, the extracellular acidification rate (ECAR), a measure of glycolytic activity, was diminished after shRNA-Blimp-1 intervention (Fig. 5E), reflecting lower basal and maximum glycolysis levels (Fig. 5F). Collectively, these findings underscore the capacity of Blimp-1 to facilitate M2 macrophage polarization through the modulation of key metabolic enzyme expressions.

### Blimp-1 modulates the metabolome of M2 macrophages

**Fig. 6:**
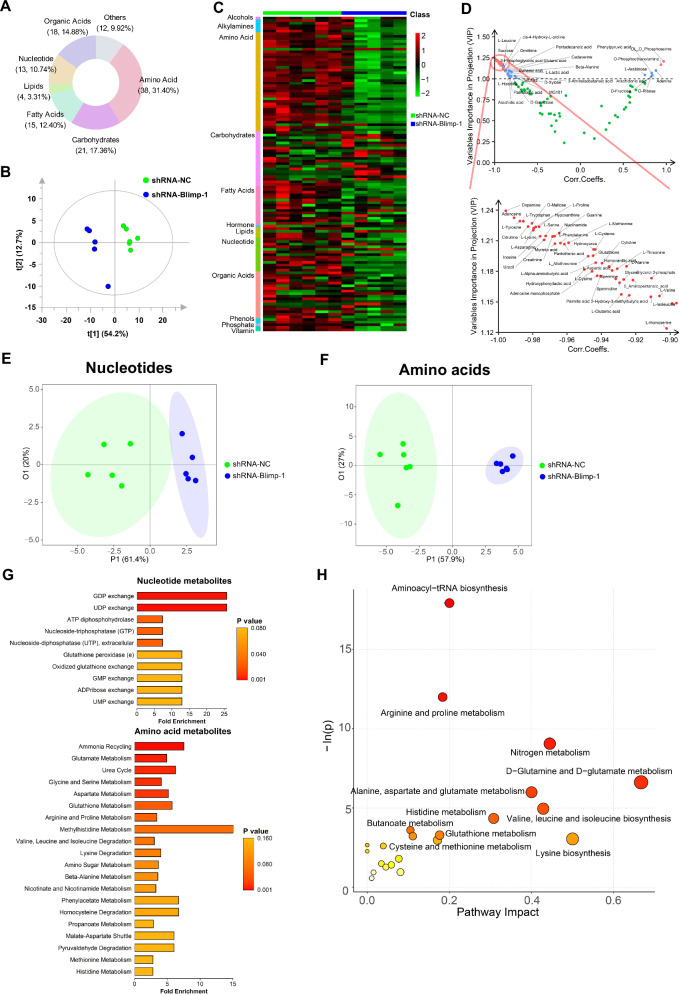
ShRNA-Blimp-1 altered metabolome in M2 macrophages. RAW264.7 cells were induced by IL-4 and IL-13 to polarize towards M2 macrophages, followed by infection with lentivirus carrying shRNA-NC or shRNA-Blimp-1. **A** Classification of the 121 metabolites identified in non-targeted metabolome analysis. **B** OPLS-DA Model Discrimination for non-targeted metabolome. **C** Heatmap of all metabolites for quantitative identification in the non-targeted metabolome. The color scale indicates the level of metabolite accumulation, while the class indicates the metabolites. **D** VIP scores comparing shRNA-Blimp-1 and shRNA-NC groups in non-targeted metabolome analysis. The green dots represented metabolites with VIP < 1.0 & |Corr.Coeffs. | < 0.9; the blue dots represented metabolites with VIP ≥ 1.0, and 0.9 ≤ |Corr.Coeffs.| < 1.0; the red dots represented metabolites with VIP ≥ 1.0, and |Corr.Coeffs.| ≥ 1.0. **E** OPLS-DA Model Discrimination for nucleotide-targeted metabolome. **F** OPLS-DA Model Discrimination for amino acid-targeted metabolome. **G** SMPDB metabolic pathway enrichment analysis of the differential metabolites in the nucleotide-targeted metabolome (*P* < 0.05). **H** KEGG metabolic pathway enrichment analysis of the differential metabolites in the amino acid-targeted metabolome (*P* < 0.05).

We furtherly conducted a non-targeted metabolomics analysis on RAW264.7 cells with Blimp-1 knockdown and identified a total of 121 metabolites (Fig. 6A), involving amino acids, carbohydrates, lipids, fatty acids, nucleotides, organic acids, and others. An orthogonal partial least squares discriminant analysis (OPLS-DA) model clearly distinguished the metabolite profiles between the shRNA-Blimp-1 and shRNA-NC groups (Fig. 6B). The heatmap revealed significant differences in amino acids and nucleotides between the two groups (Fig. 6C). The differential metabolites were displayed in Fig. 6D & Table S1, with those exceeding a variable importance in projection (VIP) scores of 1.0 considered significant (blue and red dots). Notably, the levels of Ornithine and multi-amino acids increased following Blimp-1 inhibition (Fig. 6D, above panel).

Subsequently, we conducted a quantitative metabolomics analysis targeting nucleotide metabolism and amino acid metabolism and identified 9 differential nucleotide metabolites and 17 differential amino acid metabolites between groups (VIP ≥ 1.0 & false discovery rate [FDR] < 0.05; Table S2). OPLS-DA clearly distinguished the nucleotide and amino acid metabolite of shRNA-Blimp-1 and shRNA-NC groups (Fig. 6E, F). Metabolic pathway enrichment analysis using the Small Molecule Blimp-1 knockdown significantly impacted GDP exchange, UDP exchange and ATP diphosphate hydrolase in M2 macrophages (Fig. 6G), as well as ammonia recycling, glutamine metabolism, urea cycle and glycine and serine metabolism (Fig. 6H). These findings suggest that Blimp-1 influences the metabolic pathways of nucleotides and amino acids in M2 macrophages.

### Blimp-1 enhances purine biosynthesis and the downstream Ornithine cycle in M2 macrophages

**Fig. 7:**
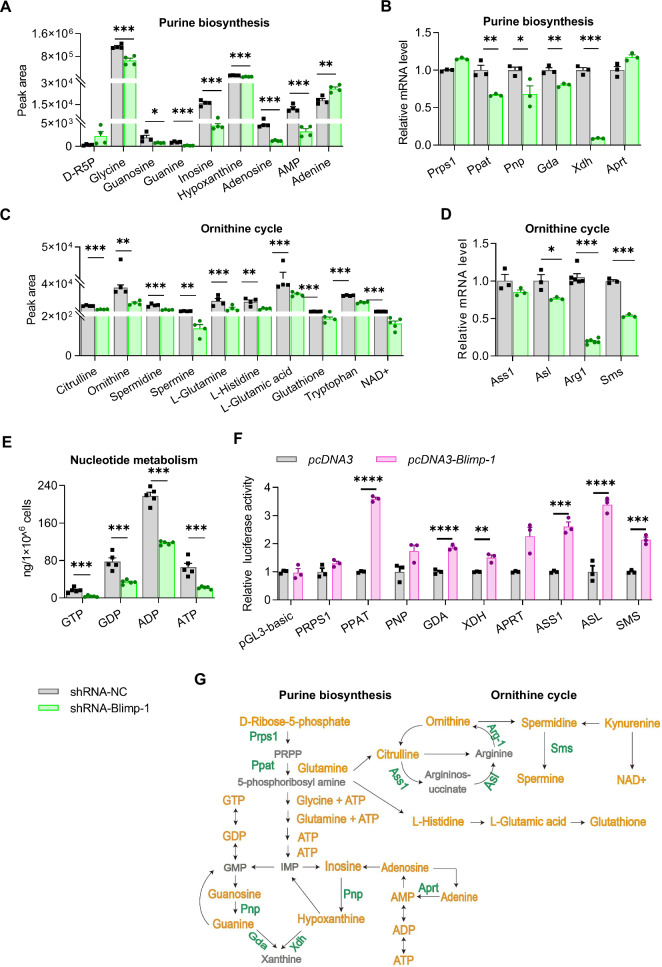
Blimp-1 regulated purine biosynthesis and the Ornithine cycle in M2 macrophages. RAW264.7 cells were induced by IL-4 and IL-13 to polarize towards M2 macrophages, followed by infection with lentivirus carrying shRNA-NC or shRNA-Blimp-1. **A** Differential abundance of metabolites in the purine biosynthesis pathway. **B** The mRNA levels of key metabolic enzymes in purine biosynthesis pathway. **C** Differential abundance of metabolites in the Ornithine cycle. **D** The mRNA levels of key metabolic enzymes in the Ornithine cycle. **E** Differential abundance of nucleotides GTP, GDP, ATP, and ADP. **F** Dual-luciferase reporter assay of metabolic enzyme gene promoters in 293 T cells. The cells were transfected with *Blimp-1* expressing plasmid or the vehicle, along with reporters of *PRPS1*, *PPAT*, *PNP*, *GDA*, *XDH*, *APRT*, *ASS1*, *ASL*, and *AMS* promoters, respectively. **G** Diagram of the differential metabolic pathway in M2 macrophages. Green indicates enzymes with upregulated expression by Blimp-1; yellow indicates metabolites with increased abundance by Blimp-1. Data are presented as the mean ± standard error of independent experiments. *P < 0.05, **P < 0.01, ***P < 0.001, ****P < 0.0001.

Building on the differential metabolite analysis, we concentrated on purine biosynthesis, nucleotide metabolism, and the Ornithine cycle. Blimp-1 knockdown led to a down-regulation of most metabolites involved in purine biosynthesis (Fig. 7A). The expression levels of genes encoding key enzymes in this pathway, such as *Ppat*, *Pnp*, *Gda*, and *Xdh*, were significantly reduced with Blimp-1 inhibition (Fig. 7B). Following Blimp-1 knockdown, the content of metabolites in the Ornithine cycle and the mRNA levels of key enzymes were significantly diminished (Fig. 7C, D). Additionally, the concentrations of nucleotide metabolites GDP, GTP, ATP, and ADP were markedly reduced (Fig. 7E). Blimp-1 directly enhances the transcriptional activity of promoters for *PPAT*, *GDA*, *XDH*, *APRT*, *ASS1*, *ASL*, and *SMA* (Fig. 7F). Collectively, these results demonstrated that Blimp-1 regulates purine biosynthesis and the downstream Ornithine cycle by promoting the transcription of key enzyme genes in M2 macrophages (Fig. 7G).

### Purine biosynthesis and Ornithine cycle metabolic regulation is involved in M2 macrophage polarization promoted by Blimp-1

**Fig. 8:**
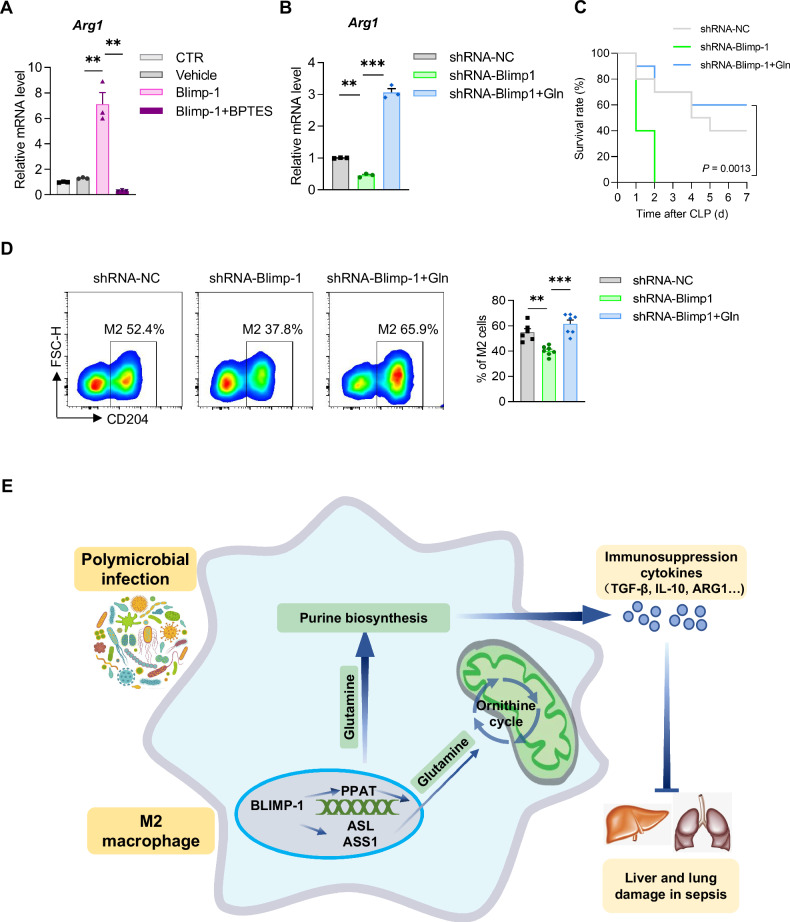
Purine biosynthesis and Ornithine cycle metabolic regulation is involved in Blimp-1-mediated M2 macrophage polarization. **A** The mRNA level of *Arg1* in BMDM transfected with *Blimp-1* plasmid or vehicle and subsequently treated with BPTES. **B** The mRNA level of *Arg1* in BMDM infected with lentivirus carrying shRNA-NC or shRNA-Blimp-1, and treated with glutamine (Gln). **C** The survival rates of CLP mice intraperitoneally injected with macrophage-targeted AAV carrying shRNA-NC or shRNA-Blimp-1, and treated with Gln, respectively (*n* = 10 per group). **D** Percentage of M2 macrophages in PLF cells. **E** A proposed schematic diagram of the regulatory mechanism of Blimp-1 on the metabolism, polarization, and function in macrophages, and its protective effect in sepsis. When the body is suffered from polymicrobial infection, the Blimp-1 expression in macrophages will be enhanced and resulted in promoting M2 polarization, as well as invigorating purine biosynthesis and the downstream Ornithine cycle through Glutamine pathway, which furtherly suppressed secretion of the inflammatory cytokine, and finally protecting the organs (liver and lung) from damage in sepsis. Data are presented as the mean ± standard error of independent experiments. *P < 0.05, **P < 0.01, ***P < 0.001, ****P < 0.0001.

We further investigated the potential role of purine biosynthesis and Ornithine cycle metabolic regulation in Blimp-1’s promotion of M2 macrophage polarization. As glutamine serves as a hub of purine biosynthesis and the Ornithine cycle, thus linking the metabolism of two distinct biological macromolecules, we transfected a plasmid expressing Blimp-1 in BMDM, and we observed a significant decrease in the mRNA levels of the M2 macrophage marker *Arg1* in BMDM post-transfection with Blimp-1, following administration of BPTES, an inhibitor of the glutamine metabolism pathway (Fig. 8A). Conversely, the mRNA levels of *Arg1* in BMDM transfected with shRNA-Blimp-1 was found to be elevated upon glutamine supplementation (Fig. 8B). Additionally, glutamine supplementation was shown to rescue the mortality of CLP mice post intervention with macrophage-targeted AAV shRNA-Blimp-1 (Fig. 8C), along with an increased proportion of M2 macrophages (Fig. 8D). A schematic diagram summarizing our proposed regulatory mechanism of Blimp-1 on the metabolism, polarization, and function in macrophages, along with its protective effects in sepsis, is presented in Fig. 8E. Taken together, these results suggest that play key roles in Blimp-1’s influence on M2 macrophage polarization and mediate Blimp-1’s impact on septic mice.

## Discussion

A central component of sepsis pathophysiology is the polarization of macrophages. Macrophages undergo distinct polarization states (M1 and M2) that significantly influence sepsis outcomes. Nrf2 has been shown to play a protective role in sepsis-induced pulmonary injury and inflammation by modulating autophagy and NF-κB/PPARγ-mediated macrophage polarization [25]. Similarly, Krüppel-like transcription factors reduce macrophage glycolysis and inflammatory cytokine secretion by suppressing the M1 macrophage polarized phenotype [26]. Our recent work revealed that Blimp-1 inhibits the secretion of inflammatory cytokines *via* multiple toll-like receptors [16], which elucidate a novel role for Blimp-1 in macrophage polarization during sepsis.

Macrophage metabolic remodeling is essential for adapting to different polarized states and their functional changes [21]. The polarization of M2 macrophages involves alterations in several metabolic pathways, including glucose, amino acid, fatty acid, and nucleotide metabolism. Specifically, in fatty acid metabolism, PGC-1β collaborates with PPARγ to inhibit the secretion of inflammatory factors in M1 macrophages and to promote the M2 polarization [27]. The mechanism may involve PPARγ and PGC-1β directly regulating ARG1 expression, with their activation playing a crucial role in mitochondrial respiration and fatty acid oxidation in M2 macrophages [19, 20].

In glucose metabolism, TKT, a key enzyme in the pentose phosphate pathway downstream of D-fructose-1.6 diphosphate, is upregulated in M1 macrophages along with glycolysis-related enzymes; however, this does not affect PPAT-related purine synthesis or cell proliferation [28]. In amino acid metabolism, GATM and GPT2, which are involved in the glutamate pathway, facilitate glutaminolysis, leading to α-ketoglutarate accumulation and M2 polarization [29, 30]. In our study, Blimp-1 suppression led to a significant reduction in the mRNA expression of *Pparg, Ppargc1b, Tkt, Gatm*, and *Gpt2* in M2 macrophages, while Blimp-1 overexpression resulted in a marked increase in these genes. These findings suggest that Blimp-1 regulates M2 macrophage polarization by modulating key metabolic enzyme expressions.

Enhanced glycolysis during M2 macrophage polarization provides a metabolic basis for improved mitochondrial oxidative phosphorylation. M2 macrophages sustain their metabolism primarily through the tricarboxylic acid cycle and mitochondrial oxidative phosphorylation, resulting a more robust oxidative metabolic profile compared to M1 macrophages [31]. Our results demonstrated that Blimp-1 knockdown led to substantial inhibition of both basal respiration and maximum respiratory capacity in mitochondrial oxidative phosphorylation, as well as glycolytic function. These results confirm that Blimp-1 regulates both mitochondrial oxidative phosphorylation and glycolysis in macrophages.

Our results claimed that Blimp-1 enhances the transcription of PPAT, ASS1, and ASL, affecting purine biosynthesis and the Ornithine cycle in M2 macrophages. Previous studies have linked the Ornithine cycle with M2 polarization [18]. Notably, the expression of ARG1, a marker of M2 macrophages, is associated with the Ornithine cycle, while citrulline consumption is indicative of M1 polarization [32]. Although research on purine biosynthesis regulation in macrophage polarization is limited, both purine metabolism and the Ornithine cycle depend on glutamine, a star amino acid that regulates macrophage function [30, 33]. Glutamine contributes α-ketoglutaric acid to the tricarboxylic acid cycle and facilitates acetylation modification of histones through acetyl-CoA, activating α-ketoglutaric acid-dependent epigenetic regulation and thereby influencing M2 macrophage polarization [34]. Actually, glutamine is the hub of purine biosynthesis and Ornithine cycle, connecting the metabolism of two kinds of biological macromolecules. Our study not only confirmed the regulatory mechanism of glutamine metabolism by Blimp-1, but also more comprehensively mapped the characteristics of glutamine-centered purine biosynthesis and Ornithine cycle metabolism in M2 macrophages. By elucidating the transcriptional regulation mechanism of Blimp-1 on various metabolic enzymes, this study revealed the metabolic regulation of M2 polarization and the pathological mechanism of sepsis progression.

While our study provides valuable insights into the role of Blimp-1 in sepsis pathogenesis, several limitations warrant consideration. First, the study predominantly utilized animal models, necessitating validation in human samples. Second, due to the lack of suitable ChIP antibodies, the direct binding site and strength of Blimp-1 to target gene promoters cannot be evaluated in vivo. Third, longitudinal studies are needed to assess the long-term effects of Blimp-1 modulation on immune function and host recovery in sepsis.

In conclusion, our study unveils a novel regulatory role for Blimp-1 in macrophage polarization during sepsis, mediated through modulation of purine biosynthesis and the Ornithine cycle. These findings expand our understanding of the complex interplay between metabolism and immune responses in sepsis pathology.

## Materials and Methods

### Culture of BMDMs

Bone marrow cells were isolated from C57BL/6 male mice aged 6–8 weeks, which were euthanized via cervical dislocation. The femurs and tibia, along with lower limb muscles, were removed, sterilized in alcohol, and promptly transferred to a biosafety cabinet. After rinsing with PBS to eliminate any remaining alcohol, the lower extremity muscles were carefully stripped using scissors and a cotton cloth. The femur and tibia were fully exposed and removed, and the epiphysis on both sides were cut with scissors. The PBS buffer was extracted from the bone marrow cavity cells using a 1 mL syringe into a 15 mL centrifuge tube. The red bone marrow strips were gently blown several times until a uniform cell suspension was achieved. This was then centrifuged at 800 rpm for 5 min, and the supernatant was discarded. The cells were cultured in DMEM complete culture medium containing 10 ng/mL M-CSF (315-02, Thermo Fisher Scientific, USA) for 7 days to achieve adherent growth of M0 macrophages. Subsequently, 100 ng/mL LPS (297-473-0, Sigma-Aldrich, Germany) and 40 ng/mL INF-γ (IF005, Sigma-Aldrich, Germany) were used to induce 2-day polarization into M1 macrophages. Alternatively, 40 ng/mL IL-4 (SAB1402819, Sigma-Aldrich, Germany) and 20 ng/mL IL-13 (I1896, Sigma-Aldrich, Germany) were used to induce 2-day polarization into M2 macrophages. A 10 mM GLS inhibitor BPTES (HY-12683, MCE, USA) was selected to inhibit the glutamine metabolic pathway, while 20 mM L-Glutamine (HY-N0390, MCE, USA) was used to supplement it. The mRNA levels of the M2 cell marker gene were measured by real-time PCR after 48 h.

### Cell culture and treatment

RAW264.7 and 293 T cells were cultured with DMEM medium (11965092, Gibco, USA) supplemented with 10% FBS (10091148, Gibco, USA) and 1% penicillin/streptomycin solution (15240112, Gibco, USA). THP-1 cells were cultured and induced to differentiate into M0 macrophages by 50 ng/mL PMA (P8139, Sigma-Aldrich, Germany) for 48 h. Differentiated THP-1 or RAW264.7 cells were transiently transfected with *Blimp-1* expressing plasmid using Lipofectamine 3000 Transfection Reagent (L3000150, Thermo Fisher Scientific, USA) for 48 h. The construction of lentiviral vector shRNA-Blimp-1 or shRNA-NC was described as our previous report [16].

### Construction of AAV

AAV particles carrying shRNA-Blimp-1 or shRNA-NC were customized by Genechem Co., Ltd. (Shanghai, China). The Blimp-1 RNAi sequence is 5’-GCCTCTTCCCTAGGTTGTATC-3’, and the scrambled NC sequence is 5’-TTCTCCGAACGTGTCACGT-3’. The shRNA was ligated into the pAAV-ApoE/hAATp-EGFP GV698 plasmid. The expressing plasmid was co-transfected with pHelper (carrying adenoviral origin genes) and pAAV-RC (carrying AAV replication and capsid genes) plasmids into AAV-293 cells for packaging recombinant AAV9 particles. The virus particles were purified through two rounds of CsCl ultracentrifugation and ultrafiltration. The shRNA-Blimp-1 AAV particles have a titer of 6.56 × 10^13 ^v.g./mL determined by real-time PCR. The concentration of AAV gDNA was detected by quantitative PCR, and the virus solution was identified by lamp test. The macrophage-targeted recombinant AAV was constructed using the pAAV-F4/80p-EGFP-MIR155(RNAi)-SV40 PolyA plasmid with the same RNAi sequence and preparation procedure, and the virus titer was 9.93 × 10^12 ^v.g./mL.

### Mice, sepsis model and in vivo intervention

All animal procedures were conducted in accordance with protocols approved by the Animal Care Research Ethics Committee of the Capital Medical University of China (AEEI-2021-050) and housed under pathogen-free conditions in a barrier facility. Male C57BL/6 mice, aged 6–8 weeks, were sourced from the Institute of Laboratory Animal Science, Chinese Academy of Medical Science (Beijing, China). A sepsis model was established using the CLP procedure, wherein the cecum was exposed, ligated at half the distance between the distal pole and the base of the cecum with a 3-0 silk suture, and punctured with an 18-gauge needle. The abdominal wall and skin were closed with 4-0 polypropylene sutures. Sterile saline (1 mL) was administered subcutaneously for fluid resuscitation during the postoperative period. A scoring system, based on three visual parameters—respiratory status, activity and response to stimuli, and eye appearance—was employed to monitor the mice over a 7-day period. The survival rate of the CLP model was about 50%, with the surviving CLP 7 d mice showing signs of recovery. Given the abdominal infection in CLP mice, we opted to intraperitoneally inject AAV particles carrying shRNA-Blimp-1 or shRNA-NC (Macrophage-targeted shRNA-Blimp-1 or Macrophage-targeted shRNA-NC) at a titer of 5.00E + 11 v.g/mL. The mice were fed for three weeks post-AAV injection before undergoing CLP surgery. Intraperitoneal injection of L-Glutamine (HY-N0390, MCE, USA 0.75 g/kg) was chosen to be completed within 24 h post-CLP surgery, with mortality observed over a 7-day period. CLP mice selected for Blimp-1 knockdown in macrophages using macrophage-targeted AAV were sampled at 18 h postoperatively.

### Splenic cell isolation

Spleen was excised and minced in PBS. Minced spleen suspension was filtered through a 70-μm filter, spun at 1200 rpm for 5 min. The cell precipitation was collected and centrifuged at 1200 rpm for 5 min. The cell precipitates were collected and split in RBC lysate (420302, BioLegend, USA) for 3 min and then rotated at 1200 rpm with PBS for 5 min. The supernatant was discarded and washed again with PBS, and an appropriate number of cells were resuspended in the FACS buffer for flow cytometry staining.

### Peritoneal lavage fluid (PLF) extraction

The abdominal skin of mice was cut open, the peritoneum was exposed, the abdominal cavity was irrigated with 5 mL 0.9% NaCl. The normal saline was sucked out after full shaking for 90 s, the supernatant was rotated at 1200 rpm for 5 min and the supernatant was discarded. The cells were resuspended with FACS buffer and stained for flow cytometry.

### Flow cytometry

Cells were washed and collected by centrifuging at 1200 rpm for 5 min, and resuspended with FACS buffer. Single cell suspensions were stained with LIVE/DEAD Zombie Green (423111, BioLegend, USA) and ɑ-CD16/CD32 (BE0307-1, BioXcell, China) before tetramer staining. The following mAbs were used: Blimp-1-PE (12-9850-82, Biosciences, USA), CD86-PE-Cy7 (60-0862, Tonbo Bioscience San Diego, USA), CD45-APC-Cy7 (109824, BioLegend, USA), CD45-eFluor™ 450 (60-0862, Tonbo Bioscience San Diego, USA), F4/80-FITC (11-4801-82, eBioscience, USA), F4/80-APC (17-4801-82, eBioscience, USA), F4/80-Alexa Fluor® 700 (123129, BioLegend, USA), CD11b-BV510 (101263, BioLegend, USA),CD11b-PerCP-Cy5.5 (65-0112, Tonbo Bioscience San Diego, USA), CD3- eFluor450 (48-0032-82, eBioscience, USA),CD3-BV421 (100228, BioLegend, USA), CD19-PE-Cy7 (115520, BioLegend, USA), CD8-FITC (MA1-10303, eBioscience, USA), CD4-Percp (100432, BioLegend, USA), CD206-APC (141708, BioLegend, USA), CD206-BV421 (141717, BioLegend, USA), Gr-1-FITC (551460, BD Bioscience, USA), CD204-PE (154709, BioLegend, USA), CD48-Percp-Cy5.5 (103422, BioLegend, USA), CD48-PE-Cy7 (103424, BioLegend, USA), Gr-1-APC (108412, BioLegend, USA), CD11c-BV605 (117333, BioLegend, USA), NK1.1-BV785 (108749, BioLegend, USA), γδT-FITC (118105, BioLegend, USA). For transcription factor Blimp-1 staining, cells were fixed and permeabilized with Fix/perm buffer (00-5523-00, Thermo Fisher Scientific, USA) prior to intracellular staining. Data acquisition and analysis were performed using a CantoII flow cytometer (BD Bioscience, USA) or SA3800 Spectral Analyzer (SONY, JPN) and FlowJo Software (version 10.1; Tree Star, USA). The flow cytometry gating strategy is as follows: M2 macrophages (CD45^+^F4/80^+^CD11b^+^CD206^+^ or F4/80^+^CD11b^+^CD204^+^), M1 macrophages (CD45^+^F4/80^+^CD11b^+^CD80^+^), neutrophils(CD45^+^Gr-1^+^CD11b^+^CD48^-^), CD4 T cells(CD45^+^CD3^+^CD4^+^CD8^-^), CD8 T cells(CD45^+^CD3^+^CD4^-^CD8^+^), B cells(CD45^+^CD3^-^CD19^+^), NK cells(CD45^+^NK1.1^+^), dendritic cells(CD45^+^CD11c^+^), and γδT cells(CD45^+^γδT^+^).

### Multiple cytokine assay

The LEGENDplexTM MU Macrophage/Microglia Panel (13-plex) and VbP Reagent (740846, BioLegend, USA) were used to detect cytokines. Add 25 μL standard product or plasma sample was added into the 96-well plate, add 25 μL/well of detection fluid and 25 μL/well detection microsphere and paste the sealing plate film. Shake for 2 h in the dark on a horizontal shaker. Centrifuge at 1,300 rpm for 5 min and discard the supernatant. Wash with 200 μL/well wash buffer, centrifuge at 1,300 rpm for 5 min, and remove the supernatant. Add 25 μL/well detection antibody reagent and shake vigorously for 1 h at room temperature, avoiding light. Add 25 μL SA-PE reagent to each well and shake vigorously for 30 min at room temperature, avoiding light. Wash twice, add 150 μL/well was buffer, fully suspended the microspheres, transfer to the flow tube for machine detection. Duplicate the flow data and enter the BioLegend website, generate the standard curve and perform data analysis and processing.

### Serum alanine aminotransferase and aspartate aminotransferase measurement

Mouse serum samples were collected and the activities of serum aspartate transaminase (AST) and alanine transaminase (ALT) were determined using an automatic analyzer (Model 7600 Series, Hitachi, Japan).

### Hematoxylin-eosin (H&E) staining

After the mice were sacrificed, liver and lung samples were separated and fixed with 4% paraformaldehyde (BL539A, Biosharp, China) for 24 h, subsequently embedded in paraffin, sliced (4–5 μm). Histological sections of tissues were subjected to H&E staining. For each tissue section, 5 to 7 visual fields were randomly chosen for scoring. The Ishak score was employed to assess the degree of inflammation and necrosis in liver H&E staining. Lung tissues were graded on a scale from 0 to 4 based on inflammation, edema, hemorrhage, and alveolar septum thickening. The final score represented a comprehensive evaluation of all four injury parameters.

### RNA isolation, reverse transcription, and real-time PCR

Total RNA was extracted with RNA extraction Kit (ET101-01-V2, Transgenbiotech, China) and reverse transcribed using the PrimeScript™ RT reagent Kit (RR047A, TaKaRa, Japan). Amplification was performed using the Power SYBR^®^ Green PCR Master Mix (4367659, Thermo Fisher Scientific, USA). The relative expression level of each transcript was normalized to murine GAPDH. The primers are listed in Table S3. The relative expression levels of target genes were calculated by 2-ΔΔCT (Livak method). Calculation formula: ΔCt = Ct target gene -Ct reference gene; ΔΔCt = ΔCt experimental group - ΔCt control group.

### Plasmid construction, transfection, and dual-luciferase assay

The promoter cDNA sequences of PRPS1, PPAT, PNP, GDA, XDH, APRT, ASS1, ASL, and SMS were constructed using the pGL3-basic-Luc-wt plasmid. Blimp-1 cDNA was cloned into pcDNA3.1+ plasmid as previous report [35]. Appropriate concentration of 293 T cells was inoculated into a 24-well plate, with each well containing 1 × 10^5^ cells. The total DNA transfected into each 24-well plate was 500 ng. Solution A consisted of 50 μL Opti-MEM medium, including 245 ng of pGL3-basic luciferase reporter carrying indicated gene promoter, 245 ng of pcDNA-BLIMP1, and 10 ng pRL-TK plasmid expressing Renilla luciferase (internal reference). Solution B was prepared by mixing 1 μL PEI-40K transfection reagent (G1802, Servicebio, China) with 49 μL Opti-MEM medium (31985062, Gibco, USA), and allowed to stand for 5 min. Solution B was then added to Solution A, mixed thoroughly, and incubated at room temperature for 15 min. The cell medium in the 24-well plate was replaced with Opti-MEM medium, and the AB mixture was then added to the respective well. This was incubated in a cell incubator at 37 °C for 4–6 h, after which the medium was replaced with DMEM complete medium. After 24 h, cell lysate was collected and detected using the Dual-Luciferase® Reporter Assay System (E1910, Promega, USA). The microplate luminescence detector was used for detection (Turner Biosystems, USA) was used for detection. The calculation formula is as follow: Normalized value = Firefly luciferase reading value (reporter gene)/Renilla luciferase reading value (internal reference gene). Relative expression ratio=experimental group normalized value/control group normalized value.

### Seahorse XF glycolytic stress and mitochondrial stress test

Mitochondrial respiratory capacity and glycolytic function of M2 macrophages were measures using Seahorse XF Glycolysis Stress Test Kit (103020-100, Agilent, USA) and Seahorse XF Cell Mito Stress Test Kit (103015-100, Agilent, USA) on a Seahorse XFe24 Analyzer (Agilent, USA). Inoculate cells at 1 × 10^4^/well, culture overnight in a CO_2_-free cell incubator at 37 °C, and the coverage of cells before loading should reach about 80–90%. Add 200 μL/well ddH_2_O to the hydration plate, and incubate the whole probe plate overnight in a CO_2_-free cell incubator at 37 °C. Discard the sterile distilled water in the hydration plate, and add 200 μL XF calibration solution to each hydration plate well. Hydrate the whole probe plate for 1 h in a CO_2_-free cell incubator at 37 °C. Prepare Seahorse test solution: 97 mL pH 7.4 Seahorse XF DMEM medium, 1 mL pyruvate (1 mM), 1 mL glutamine (2 mM) and 1 mL glucose (10 mM). Incubate the prepared test solution in a CO_2_-free cell incubator at 37 °C. Add 160 μL detection solution to cell culture wells, and place it in a CO_2_-free cell incubator for 1 h. Preheated drugs are sequentially added to the probe plate dosing holes, preparing for machine testing. Run the XF experiment on the computer, open the suite template corresponding to the wave software, set up the experimental grouping, and collect data for analysis after the experiment is completed.

### Non-targeted and targeted metabolomics analysis

The preparation of cell sample was consistent for non-targeted and targeted metabolomics detection. Briefly, 1 × 10^7^ RAW264.7 cells were centrifuged at 4 °C at 200–1000 rcf for 10 min. The cell pellets were lysed ultrasonically and centrifuged at 18,000 g for 20 min. Ten microliter supernatants were mixed with 70 μl borate buffer and 20 μl 6-minoquinolyl-N-hydroxysuccinimidyl carbamate derivatization reagent. The mixture was shaken at 1200 rpm for 10 min, then added 900 μl ultrapure water and mixed well. Using liquid chromatography mass spectrometry (LC-MS) and Metabo-Profile’s self-built database, non-targeted metabolomics analysis of metabolites in samples was performed qualitatively and quantitatively. The target metabolomics of amino acids and nucleotides in the samples were subjected to ultra-performance liquid chromatography/tandem mass spectrometry (UPLC-MS/MS) assay for quantification.

XploreMET software (V3.0, Metabo-Profile, Shanghai, China) was used for quality control of samples and annotation of metabolites in non-targeted metabolomics profiling. In both non-targeted and targeted metabolomics profiling, we firstly conducted the principal component analysis to assess the overall distribution of samples and to verify the consistency of the analytical process. Subsequently, OPLS-DA and univariate analysis (Student t-test or Mann-Whitney test) were utilized to discern the variations in metabolic profiles and to identify metabolites with differential expression among groups. We identified differentially expressed metabolites according to VIP score ≥ 1.0 in the non-targeted metabolomics, while VIP ≥ 1.0 and FDR < 0.05 in targeted metabolomics. The *P* values were adjusted with FDR method in targeted metabolomics analysis. The VIP score of each metabolite comparing shRNA-Blimp-1 and shRNA-NC groups was displayed with “ggplot2” package. The heatmap of all metabolites was drawn by “pheatmap” package. The SMPDB and KEGG metabolic pathway enrichment analysis of the differential nucleotide- and amino acid-metabolites was performed with MetaboAnalyst tool.

### Statistics analysis

A two-sided *P* value < 0.05 was considered significant. All statistical analyses were performed using GraphPad Prism 9.0 software. The Kolmogorov-Smirnov test was used to inspect the normality and homogeneity of variance of all data. For two-group comparison, *P* values were derived from the Student t-test to determine differences between groups with normally distributed data and Mann-Whitney nonparametric test with other data.

## Supplementary information

Supplemental Tables and Figures legends

Supplemental Figures

## Data Availability

The data that support the findings of this study are available from the corresponding author upon reasonable request.
