# Supplementary material for: Blimp-1 orchestrates macrophage polarization and metabolic homeostasis via purine biosynthesis in sepsis
Source: Cell Death Dis. 2025 Feb 6;16(1):72. doi: 10.1038/s41419-025-07405-6 (PMC11802726; doi:10.1038/s41419-025-07405-6)
Supplement: Supplementary file 1 — Supplemental Tables and Figures legends [file 41419_2025_7405_MOESM1_ESM.docx]

**Supplemental Tables**

**Table S1. The top twenty differential expressed metabolites identified by OPLS-DA in non-targeted metabolomics profiling.**

| Metabolite | Class | Pathway | Corr. Coeffs | VIP |
| --- | --- | --- | --- | --- |
| Dopamine | Phenols | Catecholamine Biosynthesis, Tyrosine Metabolism | 0.99574 | 1.239 |
| Adenosine | Nucleotide | Purine Metabolism | 0.99089 | 1.233 |
| L-Tyrosine | Amino Acid | Catecholamine Biosynthesis, Phenylalanine and Tyrosine Metabolism, Transcription/Translation, Tyrosine Metabolism | 0.98750 | 1.229 |
| L-Tryptophan | Amino Acid | Transcription/Translation, Tryptophan Metabolism | 0.98548 | 1.229 |
| Citrulline | Amino Acid | Arginine and Proline Metabolism, Aspartate Metabolism, Urea Cycle | 0.98274 | 1.224 |
| D-Maltose | Carbohydrates | Starch and Sucrose Metabolism | 0.98047 | 1.227 |
| L-Asparagine | Amino Acid | Ammonia Recycling, Aspartate Metabolism, Transcription/Translation | 0.97962 | 1.220 |
| L-Lysine | Amino Acid | Biotin Metabolism, Carnitine Synthesis, Lysine Degradation, Transcription/Translation | 0.97948 | 1.222 |
| L-Proline | Amino Acid | Arginine and Proline Metabolism, Transcription/Translation | 0.97828 | 1.224 |
| Hypoxanthine | Nucleotide | Purine Metabolism | 0.97629 | 1.224 |
| Inosine | Nucleotide | Purine Metabolism | 0.97569 | 1.214 |
| Uracil | Nucleotide | Beta-Alanine Metabolism, Pyrimidine Metabolism | 0.97340 | 1.211 |
| L-Serine | Amino Acid | Ammonia Recycling, Glycine and Serine Metabolism, Homocysteine Degradation, Methionine Metabolism, Sphingolipid Metabolism | 0.97252 | 1.215 |
| Creatinine | Amino Acid | NA | 0.96849 | 1.214 |
| Guanine | Nucleotide | Purine Metabolism | 0.96736 | 1.214 |
| Myristic acid | Fatty Acids | Fatty Acid Biosynthesis | 0.96704 | 1.207 |
| Niacinamide | Vitamin | Nicotinate and Nicotinamide Metabolism | 0.96459 | 1.218 |
| L-Phenylalanine | Amino Acid | Phenylalanine and Tyrosine Metabolism, Transcription/Translation | 0.96391 | 1.208 |
| Pantothenic acid | Vitamin | Beta-Alanine Metabolism, Pantothenate and CoA Biosynthesis | 0.96159 | 1.207 |
| L-Methionine | Amino Acid | NA | 0.95915 | 1.208 |

OPLS-DA, orthogonal partial least squares discriminant analysis; Corr. Coeffs., Correlation Coefficients; VIP, variable importance in projection.

**Table S2. Differential expressed metabolites in quantitative metabolomics profiling targeting amino acid metabolism and nucleotide metabolism.**

| **Class** | **Metabolite** | **Corr.Coeffs.** | **VIP** | ***P* value** | ***FDR*** |
| --- | --- | --- | --- | --- | --- |
| Amino Acids | Citrulline | -0.98569 | 1.296 | 4.62E-09 | 1.39E-07 |
| Amino Acids | Kynurenine | -0.98222 | 1.291 | 1.36E-08 | 2.04E-07 |
| Amino Acids | alpha-Aminobutyric acid | -0.96988 | 1.275 | 1.85E-07 | 1.85E-06 |
| Amino Acids | Threonine | -0.96568 | 1.269 | 3.54E-07 | 2.65E-06 |
| Amino Acids | 4-Hydroxyproline | -0.96341 | 1.266 | 4.86E-07 | 2.91E-06 |
| Amino Acids | Valine | -0.93403 | 1.228 | 8.80E-06 | 4.40E-05 |
| Amino Acids | Serine | -0.92890 | 1.221 | 1.27E-05 | 5.44E-05 |
| Amino Acids | Aminoadipic acid | -0.92029 | 1.210 | 2.21E-05 | 8.30E-05 |
| Amino Acids | Glutamine | -0.91340 | 1.201 | 3.31E-05 | 0.0001 |
| Amino Acids | Glutamic acid | -0.89387 | 1.175 | 8.85E-05 | 0.0003 |
| Amino Acids | Proline | 0.87253 | 1.147 | 0.0002 | 0.0006 |
| Amino Acids | GABA | -0.86858 | 1.142 | 0.0002 | 0.0006 |
| Amino Acids | Histidine | -0.81720 | 1.074 | 0.0012 | 0.0027 |
| Peptides | Leucine | -0.81230 | 1.068 | 0.0013 | 0.0028 |
| Amino Acids | Glutathione | -0.80491 | 1.058 | 0.0016 | 0.0032 |
| Amino Acids | 5-Hydroxylysine | -0.79724 | 1.048 | 0.0019 | 0.0036 |
| Amino Acids | Methionine | -0.79435 | 1.044 | 0.0020 | 0.0036 |
| Nucleotides | ADP | -0.98917 | 1.263 | 5.93E-08 | 8.31E-07 |
| Nucleotides | ATP | -0.97048 | 1.239 | 3.21E-06 | 2.24E-05 |
| Nucleotides | UTP | -0.96030 | 1.226 | 1.04E-05 | 4.83E-05 |
| Nucleotides | GTP | -0.95718 | 1.222 | 1.40E-05 | 4.89E-05 |
| Nucleotides | NADH | -0.95285 | 1.216 | 2.04E-05 | 5.72E-05 |
| Nucleotides | NAD | -0.94125 | 1.201 | 4.85E-05 | 9.97E-05 |
| Nucleotides | GDP | -0.94085 | 1.201 | 4.98E-05 | 9.97E-05 |
| Nucleotides | UDP | -0.86083 | 1.099 | 0.0014 | 0.0024 |
| Peptides | GSSG | -0.80948 | 1.033 | 0.0045 | 0.0071 |

Corr. Coeffs., Correlation Coefficients; VIP, variable importance in projection; FDR, false discovery rate.

**Table S3.** **The primer sequences for PCR assay.**

| Gene | Primer sequence (5'-3') |
| --- | --- |
| Mu-Gapdh | Forward: AATGGATTTGGACGCATTGGT |
|  | Reverse: TTTGCACTGGTACGTGTTGAT |
| Mu-Blimp-1 | Forward: CTTCTCTTGGAAAAACGTGTGGG |
|  | Reverse: TCATATCAGCGTCCTCCATGT |
| Mu-Tnfa | Forward: CAGGCGGTGCCTATGTCTC |
|  | Reverse: CGATCACCCCGAAGTTCAGTAG |
| Mu-Il12b | Forward: TGGAGCACTCCCCATTCCTA |
|  | Reverse: GAGCTTGCACGCAGACATTC |
| Mu-Nos2 | Forward: GGAGATGGTCCGCAAGAGAG |
|  | Reverse: ACGCTGAGTACCTCATTGGC |
| Mu-Msr1 | Forward: AGTGGCAAAGAGCAGCAAGA |
|  | Reverse: TTTGCCACCAAGGCCCATTA |
| Mu-Mrc1 | Forward: GGAGTGATGGAACCCCAGTG |
|  | Reverse: ACCCTCCGGTACTACAGCAT |
| Mu-Arg1 | Forward: GTTGGAAAGTGCACTGGGAG |
|  | Reverse: CGCTCCCCAGATTTGCAGTA |
| Mu-Pparg | Forward: CTGTGAGACCAACAGCCTGAC |
|  | Reverse: TTCCATCACGGAGAGGTCCA |
| Mu-Ppargc1b | Forward: TTGTGGAAGGAGAAGGTTGGC |
|  | Reverse: AAAGCACGTGGCTGAGTCAA |
| Mu-Tkt | Forward: CTGAAGCTGGCTTCCTACCC |
|  | Reverse: GACTCGGTAGCTGGCTTTGT |
| Mu-Gatm | Forward: GGGTTTCCAAGCCTGGGTTA |
|  | Reverse: TTTTGCCTATGTCTTTGTCCCAT |
| Mu-Gpt2 | Forward: TGAAGACCGTGCTCCACAAG |
|  | Reverse: TGAAGATGAACAGCGTTGGTG |
| Mu-Prps1 | Forward: ACTTATCCCAGAAAATCGCTGAC |
|  | Reverse: CCACACCCACTTTGAACAATGTA |
| Mu-Ppat | Forward: GCGAGGAATGTGGTGTGTTTG |
|  | Reverse: TTTAGGCACTGCACTCCCATC |
| Mu-Pnp | Forward: CAACACACTGAATATCGACCTCA |
|  | Reverse: GCTTTGGGGAAAGTTGGGTATCT |
| Mu-Xdh | Forward: ATGACCGCCTTCAGAACAAGA |
|  | Reverse: TGTCACAGCAACATGATGCAA |
| Mu-Gda | Forward: ATCCATGCCCCTCAGTATGC |
|  | Reverse: TTGTTCCAAAGTAGCAAGCCG |
| Mu-Aprt | Forward: CCCTCTTGAAAGACCCGGAC |
|  | Reverse: CTGCGATGTAGTCGATCTTGC |
| Mu-Ass1 | Forward: CTCACCTGCTATTCACTGGCA |
|  | Reverse: CATCAGATCATTTCGGCCCTT |
| Mu-Asl | Forward: CTATGACCGGCATCTGTGGAA |
|  | Reverse: AGCAACCTTGTCCAACCCTTG |
| Mu-Sms | Forward: GACCTTCAGAGTTACGACAGTG |
|  | Reverse: GAACTATGGGTGGTAATCGCTTC |
| Mu-Il10 | Forward: GCCCTTTGCTATGGTGTCCT |
|  | Reverse: CTCTGAGCTGCTGCAGGAAT |
| Mu-Ido1 | Forward: TAGAGTCAGCTCCCCCAGTC |
|  | Reverse: GCCATCAGTGGGCTTCTTCT |
| Hu-MSR1 | Forward: CCGGAAGGCCAGGAAATTCT |
|  | Reverse: TTCCCAGCGATCGTCACAAA |
| Hu-MRC1 | Forward: ATGAGAACCGGGATTGCAGG |
|  | Reverse: ATTTGGGTTCGGGAGTCGTC |
| Hu-IL10 | Forward: CTCCAAGAGAAAGGCATCTA |
|  | Reverse: CTTCATTGTCATGTAGGCTTC |
| Hu-ARG1 | Forward: GTGGAAACTTGCATGGACAAC |
|  | Reverse: AATCCTGGCACATCGGGAATC |
| Hu-ACTIN | Forward: TGGCACCCAGCACAATGAA |
|  | Reverse: CTAAGTCATAGTCCGCCTAGAAGCA |
| Hu-Blimp-1 | Forward: TGGTGGGTTAATCGGTTTGAG |
|  | Reverse: GGGATGGGCTTAATGGTGTAG |
| Hu-Blimp-1 | Reverse: GGGATGGGCTTAATGGTGTAG |

**Supplemental Figures**

**Suppl Fig. 1. Expression of Blimp-1 in splenic immune cells.**

**(A)** Representative dot plot of flow cytometry for mouse splenic lymphocytes. **(B)** Geometric mean of Blimp-1 in splenic immune cells from sham mice and CLP mice. **(C)** Geometric mean of Blimp-1 in PLF dendritic cells, NK cells and γδT cells from sham mice and CLP mice. **(D)** Percentage of dendritic cells, NK cells and γδT cells in PLF, assessed by flow cytometry. Data are presented as the mean ± standard error of independent experiments. **P* < 0.05, ***P* < 0.01, ****P* < 0.001, *****P* < 0.0001.

**Suppl Fig. 2.** **The AAV vectors expressed in the mice.**

**(A)** The mRNA level of *Blimp-1* in the intraperitoneal lavage and spleen of CLP mice intraperitoneally injected with adeno-associated virus (AAV) harboring shRNA-NC or shRNA-Blimp-1, respectively (n = 8 per group). **(B)** GFP+ expressing cells in the peripheral blood of CLP mice that were not intraperitoneally injected with AAV and CLP mice that were intraperitoneally injected with AAV harboring shRNA-NC or shRNA-Blimp-1. **(C)** GFP expression in the liver and lung of CLP mice intraperitoneally injected with macrophage-targeted AAV containing shRNA-NC or shRNA-Blimp-1. Scale bar = 50 μm. Data are presented as the mean ± standard error of independent experiments. **P* < 0.05, ***P* < 0.01, ****P* < 0.001, *****P* < 0.0001.

**Suppl Fig. 3. Cytokine levels in the supernatant of BMDMs.**

BMDM differentiation was induced by 10 ng/mL M-CSF for 7 days, followed by M1 polarization with 100 ng/mL LPS and 40 ng/mL INF-γ for 2 days, or by M2 polarization with 40 ng/mL IL-4 and 20 ng/mL IL-13 for 2 days. **(A)** Levels of signature cytokines for M1 macrophages. **(B)** Levels of signature cytokines for M2 macrophages. Data are presented as the mean ± standard error of independent experiments. **P* < 0.05, ***P* < 0.01, ****P* < 0.001, *****P* < 0.0001.
