# Supplementary figures and images for: Blimp-1 orchestrates macrophage polarization and metabolic homeostasis via purine biosynthesis in sepsis
Source: Cell Death Dis. 2025 Feb 6;16(1):72. doi: 10.1038/s41419-025-07405-6 (PMC11802726; doi:10.1038/s41419-025-07405-6)

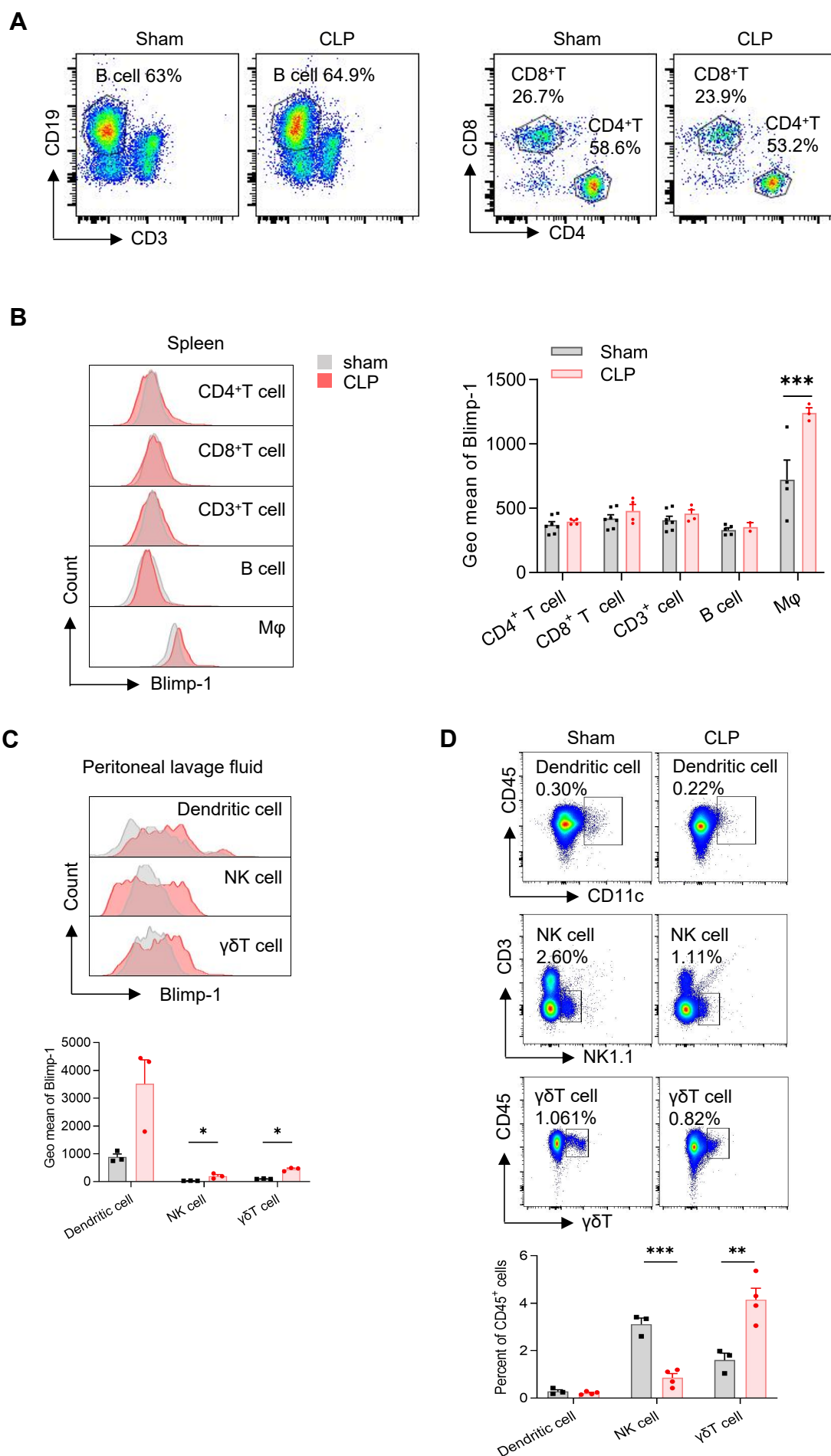

**Fig. S1**

**A**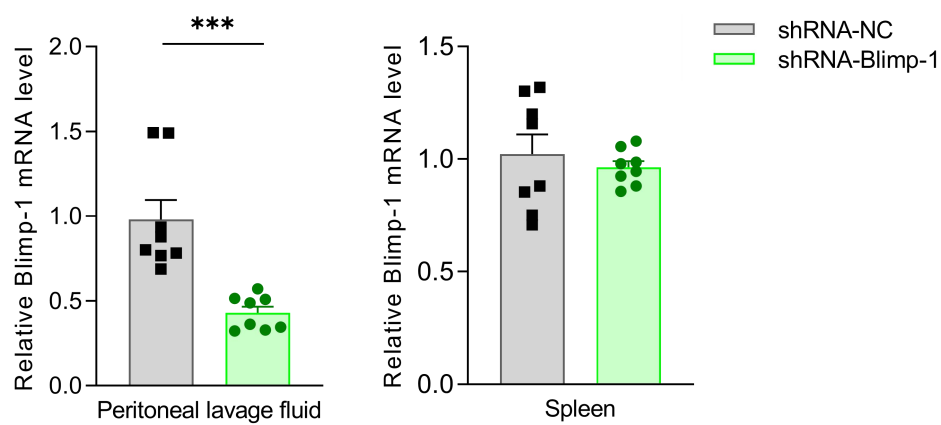**B**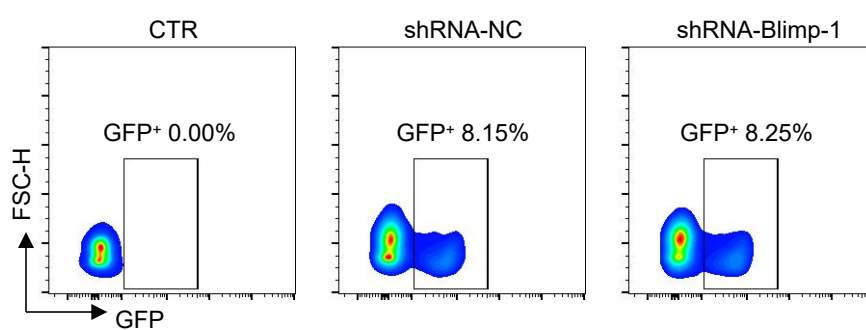**C**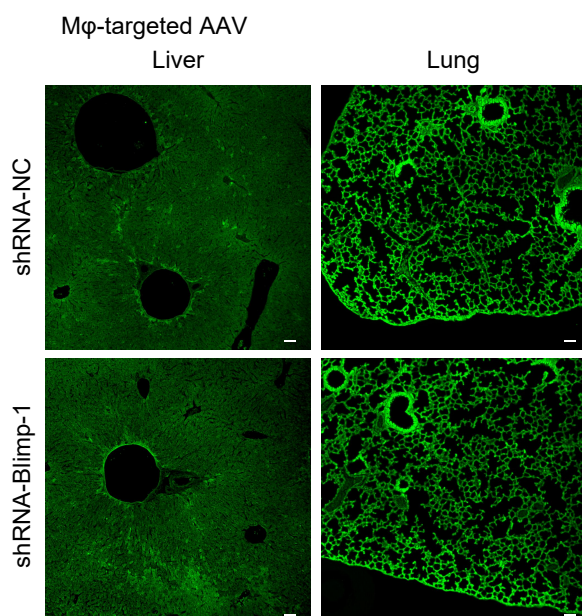

**A**

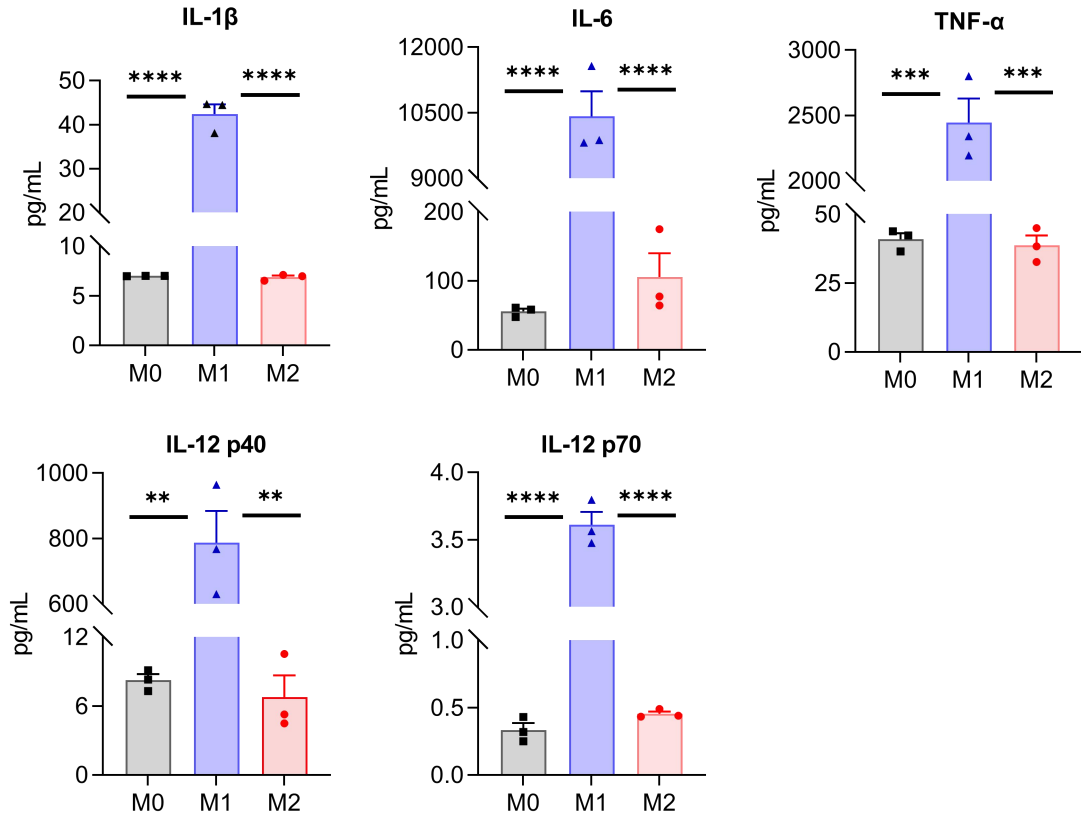

**B**

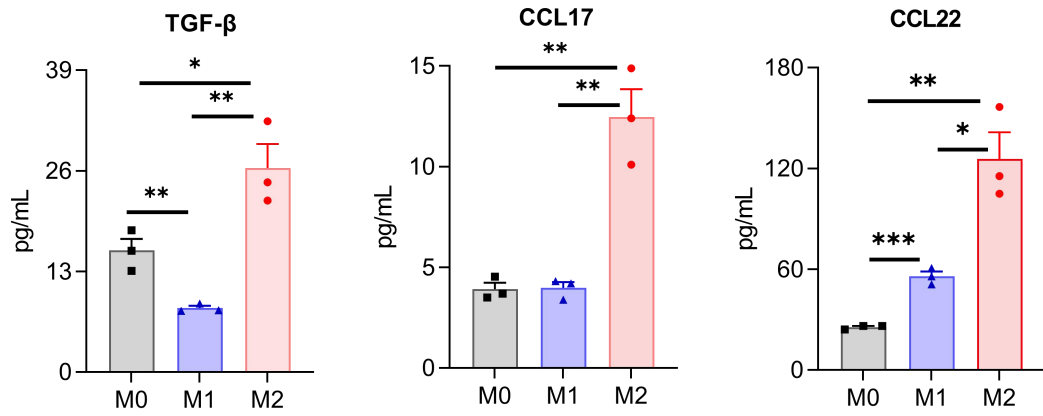

**Fig. S3**

Supplement: Supplementary file 2 — Supplemental Figures [file 41419_2025_7405_MOESM2_ESM.pdf]
